# The Smart Meter Challenge: Feasibility of Autonomous Indoor IoT Devices Depending on Its Energy Harvesting Source and IoT Wireless Technology

**DOI:** 10.3390/s21227433

**Published:** 2021-11-09

**Authors:** Edgar Saavedra, Laura Mascaraque, Gonzalo Calderon, Guillermo del Campo, Asuncion Santamaria

**Affiliations:** CeDInt-UPM, Universidad Politécnica de Madrid, Campus de Montegancedo, Pozuelo de Alarcón, 28223 Madrid, Spain; lmascaraque@cedint.upm.es (L.M.); gcalderon@cedint.upm.es (G.C.); gcampo@cedint.upm.es (G.d.C.); asun.santamaria@upm.es (A.S.)

**Keywords:** smart meter, Internet of Things, energy harvesting, energy efficiency, LPWAN, Sigfox, LoRaWAN, NB-IoT, Wi-Fi, BLE, IoT, IIoT

## Abstract

Most smart meters are connected and powered by the electric mains, requiring the service interruption and qualified personnel for their installation. Wireless technologies and energy harvesting techniques have been proved as alternatives for communications and power supply, respectively. In this work, we analyse the energy consumption of the most used IoT wireless technologies nowadays: Sigfox, LoRaWAN, NB-IoT, Wi-Fi, BLE. Smart meters’ energy consumption accounts for metering, standby and communication processes. Experimental measurements show that communication consumption may vary upon the specific characteristics of each wireless communication technology—payload, connection establishment, transmission time. Results show that the selection of a specific technology will depend on the application requirements (message payload, metering period) and location constraints (communication range, infrastructure availability). Besides, we compare the performance of the most suitable energy harvesting (EH) techniques for smart meters: photovoltaic (PV), radiofrequency (RF) and magnetic induction (MIEH). Thus, EH technique selection will depend on the availability of each source at the smart meter’s location. The most appropriate combination of IoT wireless technology and EH technique must be selected accordingly to the very use case requirements and constraints.

## 1. Introduction

In the last few years, the number of IoT devices has grown significantly, so does the spending in IoT-related fields, which is expected to increase by 24% in 2021 [1]. Moreover, the global IoT market will be worth more than 1200 billion (×10^9^) euros by 2027 [2]. It is estimated that the IoT was born in 2008, with the things-to-people ratio growing from 0.08 in 2003 to 1.84 in 2010 [3], and leading to almost 4.5 in 2021, with 35 billion devices connected [4], currently adding more than 120 new devices per second.

IoT devices may be used for a wide range of applications: healthcare, ambient conditions monitoring, workout tracking, infrastructures monitoring, failure prevention, smart metering, etc. The latter is the one we focus on in this work. Following our research presented in [5], in which an autonomous Sigfox smart meter was presented, in this paper, we analyse the feasibility of indoor IoT nodes. Specifically, we focus on smart meters with different energy harvesting (EH) sources and wireless technologies—although it might be extrapolated to any kind of indoor sensor.

Regarding the smart meter field, most of them are directly connected to the mains—such as those installed by electric companies—[6,7,8], so an intrusion in the electrical panel is required for installation, which leads to the requirement of a specialist and a temporary interruption in power supply. All these cons increase costs and prevent new deployments [9,10].

Considering the great growth in the IoT industry, the ability to keep smart meters autonomous, adapting their working conditions by their scenario necessities is crucial to encourage companies—and people—to adopt smart meters. Thus, we are about to compare the main trendy wireless communication technologies workable in smart meters—Sigfox, LoRaWAN, NB-IoT, Wi-Fi, BLE—for an extensive variety of scenarios. At the same time, we compare the most adequate EH sources for indoor wireless sensors: photovoltaic (PV), radiofrequency (RF); and specifically for smart meters: magnetic induction (MIEH). Based on metering application requisites and smart meter location constraints, this work aims to help smart meters’ developers and adopters with a roadmap to select the most suitable wireless communication technologies and energy harvesting techniques.

The rest of the paper is organised as follows: Section 2 describes the smart meter under consideration for this review, characterising both its consumption and working principle. Section 3 assesses the actual electrical consumption for each wireless technology in the spotlight. In Section 4, we sum up the existing literature to provide data on EH generations for every technique. Finally, in Section 5, the feasibility of different combinations between IoT technologies and EH sources are discussed, supported by a brief conclusion in Section 6.

## 2. Targeted Smart Meter

**Figure 1:**
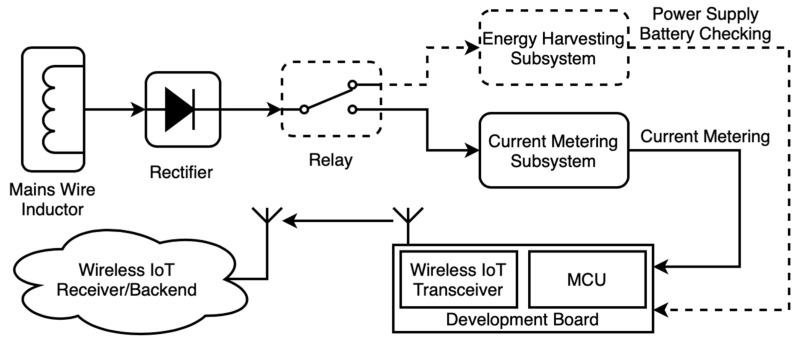
General block-diagram of the smart meter under consideration.

The smart meter used in the development of this work is inspired by the one we developed in [5], except for the EH Subsystem (see Figure 1), which is removed and characterised accordingly as EH source in Section 4. In this section, we will briefly explain the main characteristics of the smart meter.

### 2.1. Working Principle

**Figure 2:**
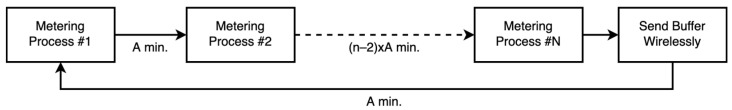
Graphical representation of the main behaviour of the smart meter.

The working principle of the smart meter is that it is event-driven, with a variable metering period (*A* minutes) depending on the working conditions. Current measurements are stored in a buffer (sized to *N* measurements) and then sent when the buffer is full—see Figure 2. One measurement is 1 byte in size, as it is encoded in tenths on amperes (dA) with 1 dA (0.1 A) precision, which would allow measurements between 0 and 255 dA (0–25.5 A). 

This buffer is sent to the corresponding wireless receiver depending on the wireless technology that we are testing. It may be a base station that sends the data to a backend (Sigfox, NB-IoT), an ad-hoc wireless gateway/host (LoRaWAN, Wi-Fi), or a receiver device scanning messages (BLE).

As well as the metering period may be changed based on the scenario conditions, so is the buffer. In the original design, the buffer was stated to 12 measurements due to Sigfox constrains [5], and the metering period was 15 min first, with latter tests of 5-min periods. 

In this work, as other wireless technologies are used, the buffer size and the period may vary and adapt to the characteristics of each wireless technology.

### 2.2. Development Board

The development used for the smart meter is a FiPy [11]. This board provides Sigfox, LoRaWAN, NB-IoT, Wi-Fi and BLE in the same device, thus making the comparison between wireless technologies easier, as the basal consumption of the board is the same with no regard to the wireless technology under study. The microcontroller (MCU) is an Xtensa dual-core 32-bit LX6, which is programmed in MicroPython.

**Figure 3:**
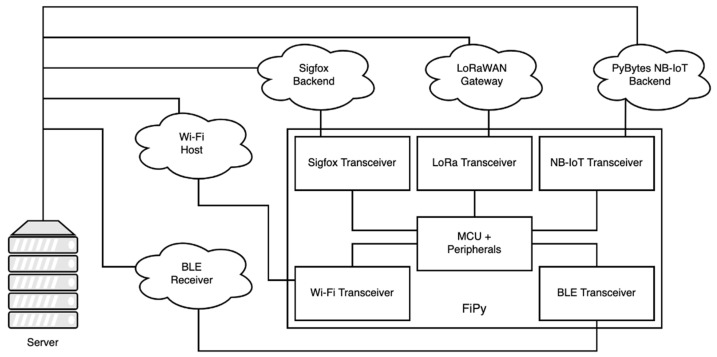
Layout for the five different wireless technologies under consideration.

Figure 3 shows the basic layout for the five different wireless technologies embedded into the development board. Being the smart meter workable across different technologies makes the server ubiquitous and interoperable between smart metering instances—be they any of the aforementioned wireless technologies.

### 2.3. Current Metering

The metering range is 0–25 A as it is an adequate range for most domestic scenarios. However, this range is easily adjusted by making small changes in the current metering subsystem [5], and it is not crucial in the object of this paper.

**Figure 4:**
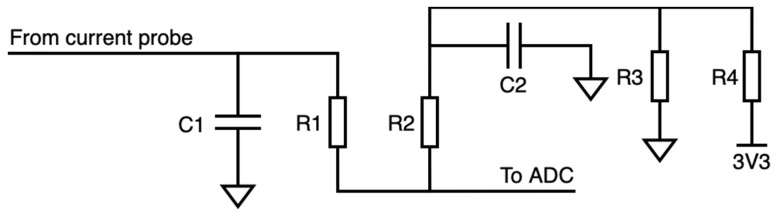
Conceptual schematic of the current metering subsystem.

The Metering Subsystem is made as one can see in Figure 4. It is based on a voltage divider to convert the incoming current-proportional signal from the current probe into an ADC-measurable voltage signal. As depicted in Figure 2, the signal outcoming the current probe is first rectified. This allows a simpler circuit design for both current metering and energy harvesting (MIEH).

Resistors were adjusted accordingly to set the proper metering range within the ADC input range.

### 2.4. Metering Consumption

**Figure A1:**
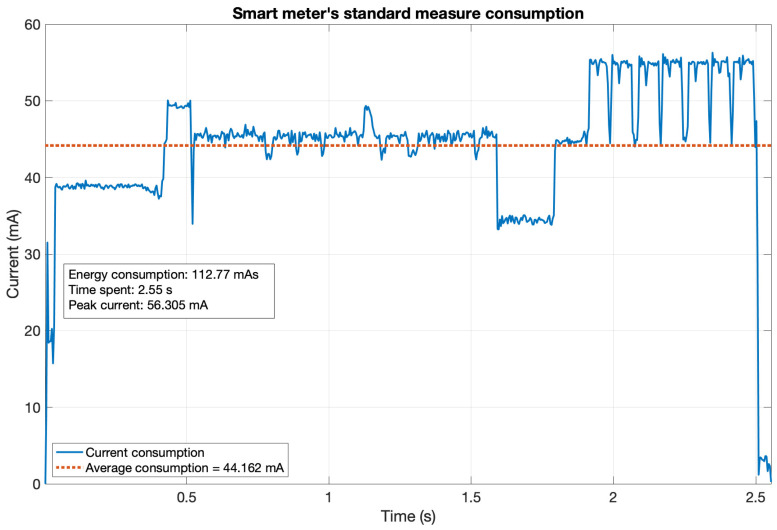
Current consumption detail of the smart meter’s metering process.

Considering the functioning principle described in Section 2.1 hereof, the specific metering—data communication apart—consumption may be: (1) that coming from every metering process, and (2) stand-by time between metering processes:

1. The consumption due to the metering process will be the same indistinctively to the wireless technology used, as no wireless transceiver is turned on for current metering and the device is always the same: 44.16 mA during 2.55 s, i.e., 112.77 mAs—see Figure A1.
2. The stand-by consumption is a constant current value that is demanded during the deep-sleep period of the device, i.e., when the device is waiting for the timer to expire, and another current measurement is about to be done—this time is *A* minutes in Figure 2: 16.28 µA during *A* min, i.e., *A* × 1.29 mAs. Datasheet [11] claims a deep-sleep current consumption of 25 µA at 3.3 V, fact that agrees with our 16.28 µA at 5 V.

These two different consumptions will compute for the energy consumption of the very smart meter. Measurements taken with the aid of a Keysight/Agilent 34410A multimeter [12] at 5 V.

In this manner, we split apart the consumption due to the wireless IoT technology—which is evaluated in Section 3 and will vary across protocols. The consumption of the wireless transmission happens eventually when the measurements buffer is full—corresponding to *#N* in Figure 2.

## 3. IoT Wireless Technologies Characterisation

In this section, the five IoT wireless technologies under study are evaluated. As the main objective of this work is to evaluate the feasibility of different wireless technologies according to their energy consumption, current consumption measurements are taken for each technology: Sigfox, LoRaWAN, NB-IoT, Wi-Fi and BLE.

We have selected these technologies as they tend to be the most used, supported, and well-known IoT wireless technologies nowadays. Furthermore, they represent a wide range of characteristics: some of them need to deploy a dedicated infrastructure (LoRaWAN, Wi-Fi, BLE) and others provide their own network for receiving messages (Sigfox, NB-IoT); Wi-Fi and BLE provide a short range whilst the others provide a very long range; they differ as well in data rate, payload, or the necessity of a handshake to send messages. These facts make the energy consumption comparison tougher as their behaviour is not equivalent. This will be discussed in Section 5. 

For consumption portrayal, message payloads from 12 bytes (Sigfox limitation) up to 768 bytes will be considered—double-fold increments. Furthermore, the consumption of the handshake or communication establishment for those technologies requiring it is considered apart from that of the transmission itself.

Then, we will evaluate the choice of using larger messages with the technologies supporting that. Several protocols provide very high data rates, which may lead to an increase in energy efficiency provided that larger messages are used, as they will deliver fast, avoiding headers and eventual handshakes. Table 1 [13,14,15] summarises some critical points for the wireless technologies under consideration—for the transceivers embedded in our development board.

Depending on the message size, a different number of transmissions will be needed, due to the maximum payload allowed by each technology for a single message. Hence, the consumptions required for each technology would be determined by the consumption of the connection establishment plus X times that of the message transmission, depending on the number of messages required—see Table 2.

**Figure 5:**
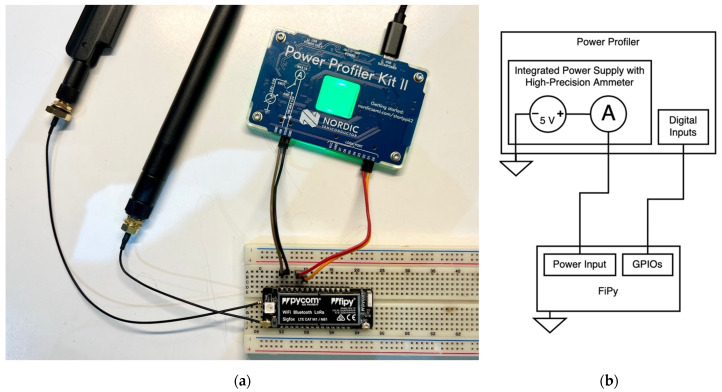
Current measurement setup: (**a**) Real picture of the Power Profiler Kit II measuring current consumption of the development board; (**b**) Block-diagram of the current measurement setup.

For current consumption characterisation, the equipment we used is a Nordic Semiconductor Power Profiler Kit II [16], which provides current sample rates of up to 100 kS/s (thousand-samples per second) with a resolution of up to 200 nA. Every current measurement is made with just the development board attached to the Power Profiler, using its embedded power source and high-precision ammeter, and only the essential peripherals turned on (see Figure 5).

So as to evaluate the actual accuracy of the Power Profiler, we set a test bed as described in Appendix B, Figure A11. The set was excited with a 100 Hz sinusoidal wave, characterised at the same time by the Power Profiler and a Keysight/Agilent 34410A multimeter—device taken as reference. Readings from the multimeter claim:

- *I*_DC_ = 92.825 mA
- *I*_AC_ = 20.539 mA_RMS_

Considering that we are working with a sinusoidal wave, its peak value—which is that happened at 100 Hz—can be calculated as in Equation (1):

(1) $$I_{100\;\mathrm{Hz}}=I_{{\mathrm{AC}}_{\mathrm{RMS}}}\times \sqrt{2}=20.539\times \sqrt{2}=29.05\;\mathrm{mA}$$

The signal measured by the Power Profiler for 10 periods is depicted in Figure A12, as well as its Fast Fourier Transform (FFT)—performed with the aid of Matlab. In the FFT plot, we can see that there are only noticeable current values for 0 Hz (DC) and 100 Hz. These values are, with their relative errors (ε):

- *I*_0 Hz_ = 90.61 mA; (ε_0 Hz_ = 2.4%)
- *I*_100 Hz_ = 28.71 mA; (ε_100 Hz_ = 1.3%)

Besides, we extracted the value of the signal in 1000 same-phase sampling points (0 radians, which corresponds to a sine wave’s nulls). For this set of values, the standard deviation was reckoned, happening to be 0.2492 mA for a mean value of 90.61 mA (0.3%).

Table 3 sums up the consumption for every technology characterised, which can be seen in detail in the following subsections and Appendix A. For the consumption characterisation, we measured the current demand for each wireless technology synchronously with the FiPy’s firmware. This was made by triggering a digital signal during the transmission process. The consumption appearing in Table 3 corresponds to an average of a cluster of 100 current measurements for each technology. Range for different measurements is less than 5%, which is an acceptable tolerance value for characterisation taking into account the accuracy of the Power Profiler.

In this table, we also depict the current demand of the development board’s transceivers for each technology according to the manufacturer (Wi-Fi/BLE [17], LoRa/Sigfox [18], NB-IoT [11]), converted to 5 V (MTC). The current demands provided by the manufacturers do not always comply with our specific working characteristics, reason why they are shown as an upper limit—nevertheless, these data are useful to check that measured consumptions are in a reasonable margin within those of the manufacturer:

- Sigfox/LoRa transceiver’s current demand is specified for +17 dBm, but not for +14 dBm, which is our case.
- NB-IoT transceiver’s current demand is only specified for a possible maximum 1.5 W power, but actual power profile would depend upon network conditions.

The idle consumption of the board was also characterised and happened to be around 37 mA. This current must be subtracted to that measured for the whole board when transmitting (Appendix A) in order to isolate the specific experimental consumption of the transceiver’s transmission (ETC), since idle current demand is due to the development board’s MCU and peripherals before wireless transceivers are activated. However, this is not the case for Wi-Fi and BLE as the transceiver is embedded into the MCU, and the consumption data provided by the manufacturer take the whole board into consideration.

The consumption coming from connection establishment (or just modem initialisation) is also depicted in Table 3. This consumption is relevant, as the device will need to do this process when waking up from deep sleep for the technologies requiring it.

In the upcoming subsections, each technology will be deeply analysed. First, the consumption for the initial 12-byte-payload message is explained; then, a table shows the measured consumptions for larger payload sizes for each technology. Detailed consumption figures are attached in Appendix A. 

### 3.1. Sigfox

The Sigfox 12-byte transmission process lasts 10.6 s with an average current consumption of 75.93 mA, which leads to an energy consumption of 804.80 mAs. Sigfox does not need a handshake for the connection to be established, so this energy is the only needed when sending a message. 

**Figure A2:**
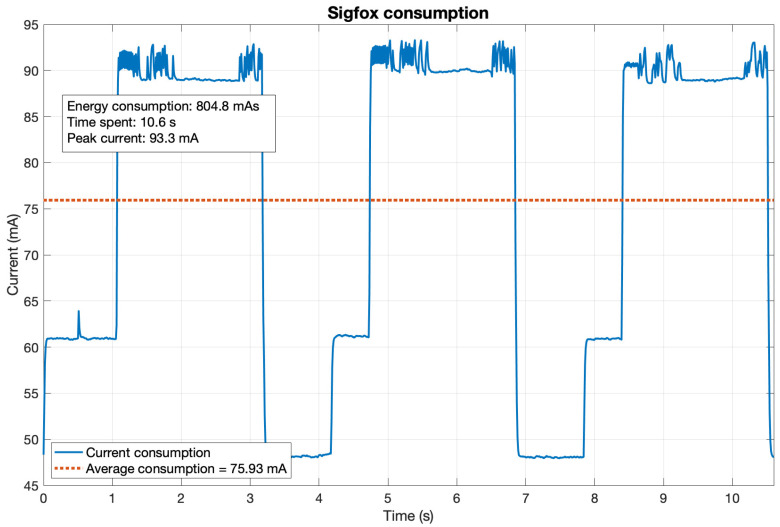
Current consumption detail for the Sigfox transmission. Data acquired at 100 s/s (samples per second).

In the consumption plot (Figure A2), it is clearly noticeable the three transmissions in three different carriers that Sigfox performs to ensure delivery: the three big waves that last about two seconds, which is the time on air of a 12-byte Sigfox message.

For larger payloads, check Table 4.

### 3.2. LoRaWAN

LoRaWAN allows different configurations regarding spreading factor (SF) and bandwidth. In this work, we assume the most used in the LoRa field is the proper one to use, with corresponds to using an SF7 with 125 kHz. Other SF could be used if messages were lost, but this configuration works well for us whilst providing the fastest data rates, which means less time on air, thus less energy spent.

**Figure A3:**
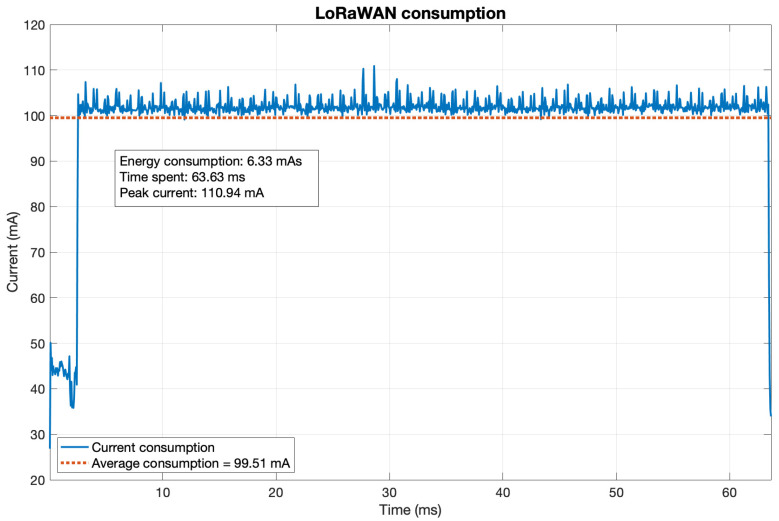
Current consumption detail for the LoRaWAN transmission. Data acquired at 100 kS/s, resampled at 1/6 for plotting.

With this set, the LoRaWAN 12-byte transmission process lasts for 63.63 ms, with an average consumption of 99.51 mA, which leads to an energy consumption of 6.33 mAs for the LoRaWAN process (see Figure A3). However, it is important to note that after transmission, the LoRa transceiver may keep activated for a while to be able to receive messages. We will not consider that phenomenon here as we truly think that it is not necessary for message transmission—the main purpose of this work is comparing only the uplink channel.

**Figure A4:**
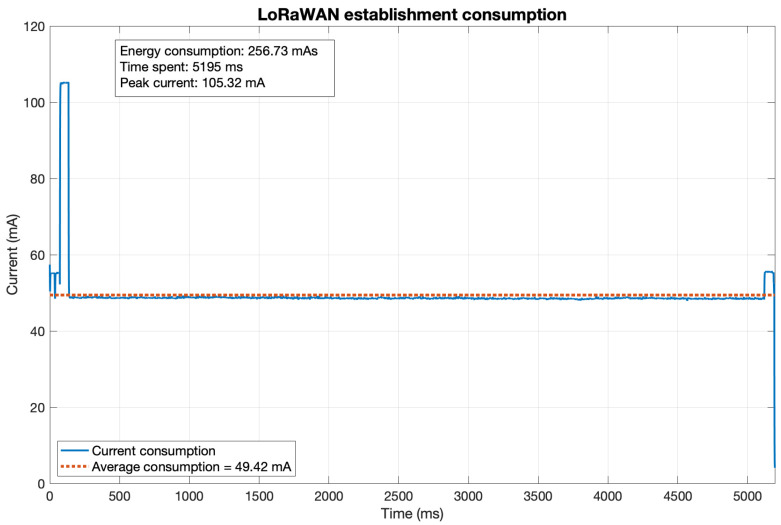
Current consumption detail for the LoRaWAN connection. Data acquired at 1 kS/s.

LoRaWAN needs connection establishment before sending messages. When the device is put in a deep sleep mode, the connection needs to be done again as the device loses its previous state. The consumption for the connection establishment can be seen in Figure A4. This process lasts 5.2 s with an average consumption of 49.42 mA, which leads to an energy consumption of 256.73 mAs.

Thus, the full energy consumption for a LoRaWAN 12-byte message is 263.06 mAs—considering both the establishment and the transmission. For larger payloads, check Table 5.

### 3.3. NB-IoT

**Figure A5:**
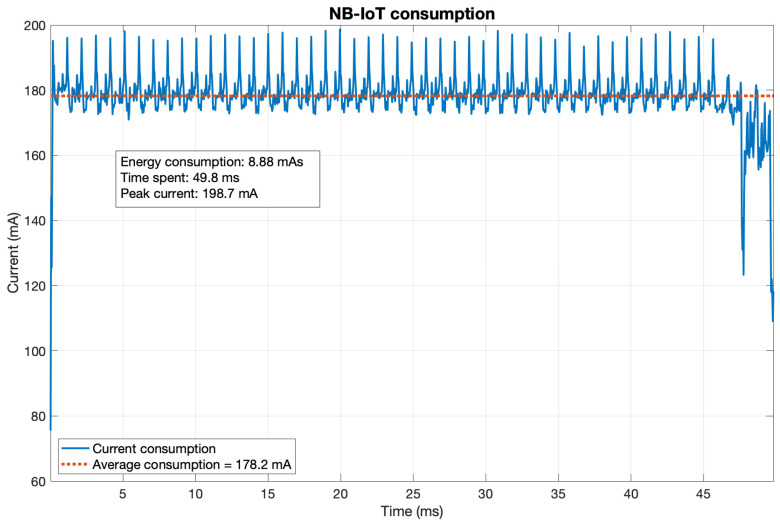
Current consumption detail for the NB-IoT transmission. Data acquired at 100 kS/s, resampled at 1/4 for plotting.

The NB-IoT 12-byte transmission process lasts for 49.8 ms, with an average current of 178.2 mA, which leads to an energy consumption of 8.88 mAs (see Figure A5). The consumption for NB-IoT does not increase much as message payload increases—as measured in Table 6. This wireless technology provides very high data rates which makes the time spent not definitely increase when we use small byte-sized increases.

**Figure A6:**
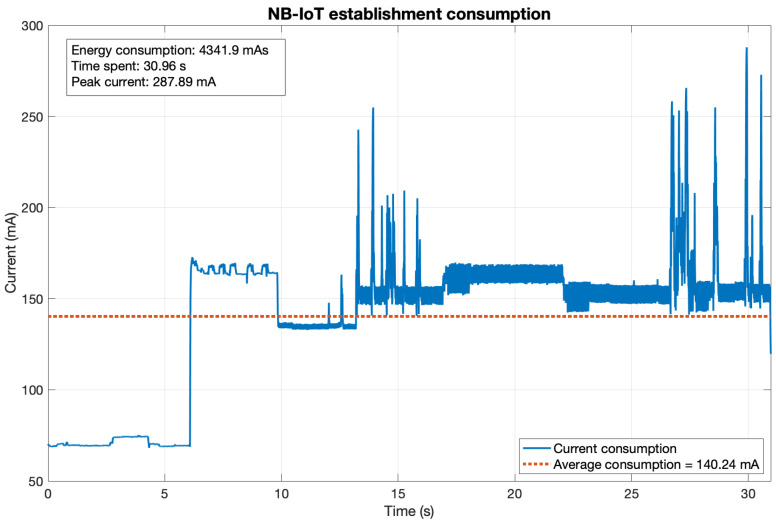
Current consumption for the NB-IoT connection establishment. Data acquired at 100 S/s.

NB-IoT takes a long time to establish the connection. The modem first needs to be initialised, then attach to the network, then connect to the service. The time needed for attaching and connecting to the network can vary depending on the NB-IoT network conditions (Vodafone Spain), so will the energy consumption. An average process is shown in Figure A6, with an energy consumption of 4342 mAs.

We can see that the consumption coming from the transmission is negligible considering that from the connection establishment (8.88 mAs vs. 4341.9 mAs), with a total of 4350.8 mAs for a 12-byte message. For larger payloads, check Table 6.

### 3.4. Wi-Fi

**Figure A7:**
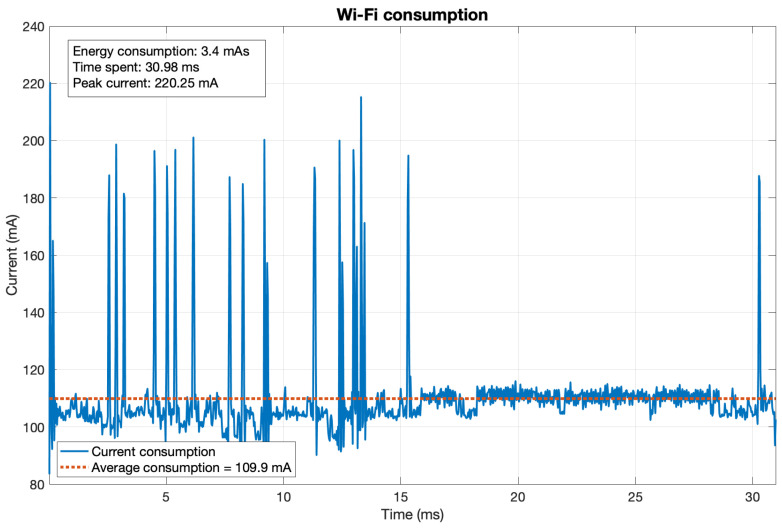
Current consumption detail for the Wi-Fi transmission. Data acquired at 100 kS/s, resampled at 1/3 for plotting.

The Wi-Fi transmission process lasts for 30.98 ms, with an average current of 109.0 mA, which leads to an energy consumption of 3.4 mAs. The Wi-Fi consumption does not depend on the packet size, as it is meant for larger messages. The time it takes for the transmission to be made is not consistent, however, as it depends on the current network traffic and network characteristics. For illustration, let us consider an average transmission as in Figure A7.

**Figure A8:**
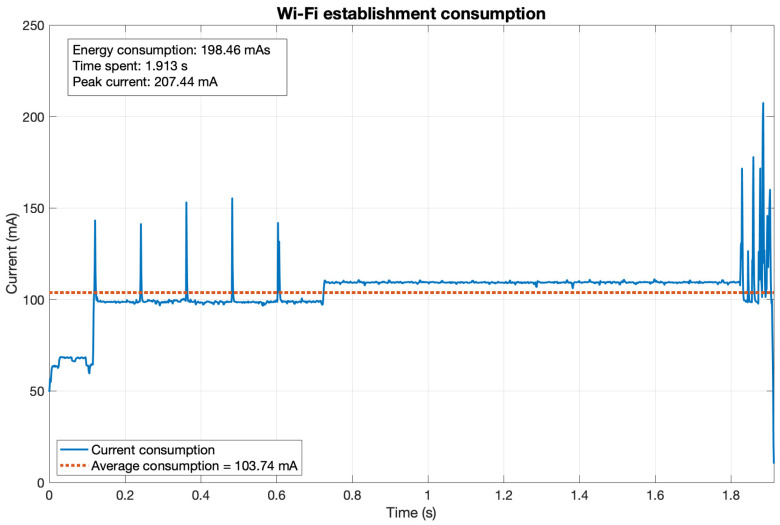
Current consumption detail for the Wi-Fi connection establishment. Data acquired at 1 kS/s.

Wi-Fi needs to establish a connection before sending messages. The modem needs to be reinitialised every time the device wakes from a sleep mode as well. This time will vary depending on real working conditions as the Wi-Fi connection time is not only dependant on the IoT device. In Figure A8, the consumption for modem initialisation and connection establishment can be seen.

Thus, the whole consumption required to send a Wi-Fi message is that coming from the establishment and the transmission per se, which is 201.86 mAs. For larger payloads, check Table 7.

### 3.5. BLE

For the case of BLE, the FiPy board still provides a limited support of the protocol. Only advertisements are supported, and no security can be implemented. Thus, we implement transmissions over BLE as advertisements that are received by another BLE device. 

Moreover, the size of message does not matter as the driver always fill the user datagram with dumb bytes. We have checked in the lab that the time and current waveform—i.e., the energy consumed—for the advertisement transmission is the same either with one user byte or the maximum 25 user bytes.

**Figure A9:**
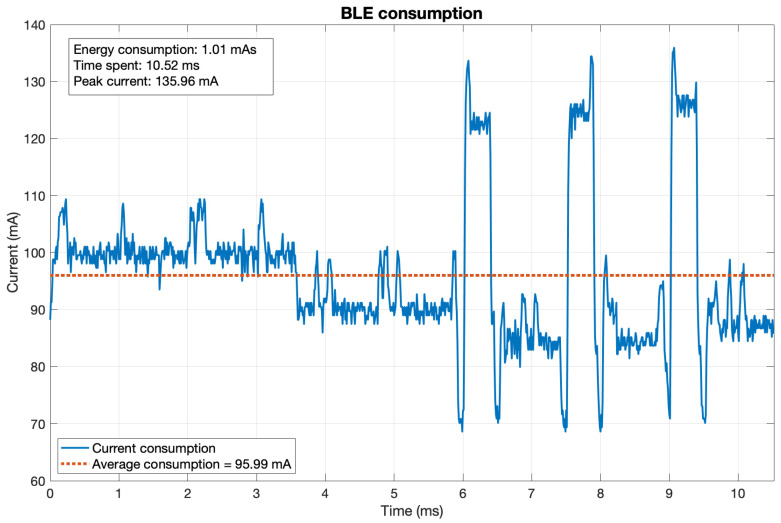
Current consumption detail for the BLE transmission. Data acquired at 100 kS/s.

In Figure A9, the current consumption for the BLE transmission process (12-byte message) can be seen. 

Since the BLE advertisement is set until the transmission finishes, the time spent is 10.52 ms, with an average consumption of 95.99 mA, which leads to an energy consumption of 1.01 mAs for the transmission of a 12-byte BLE message.

**Figure A10:**
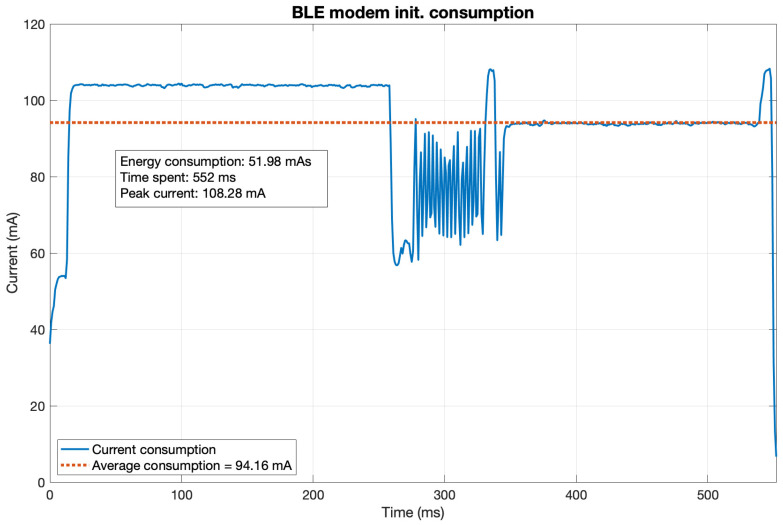
Current consumption for the BLE modem initialisation. Data acquired at 1 kS/s.

**Figure A11:**
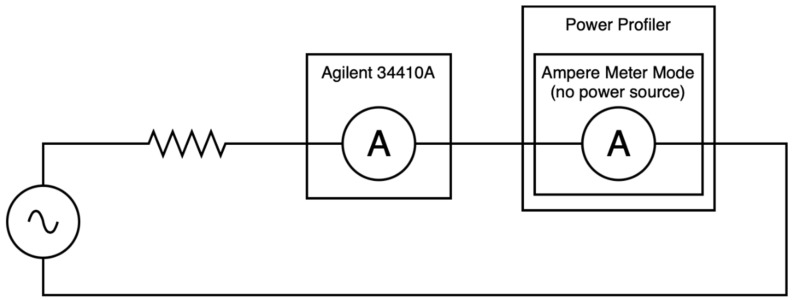
Setup used to determine the Power Profiler’s accuracy.

**Figure A12:**
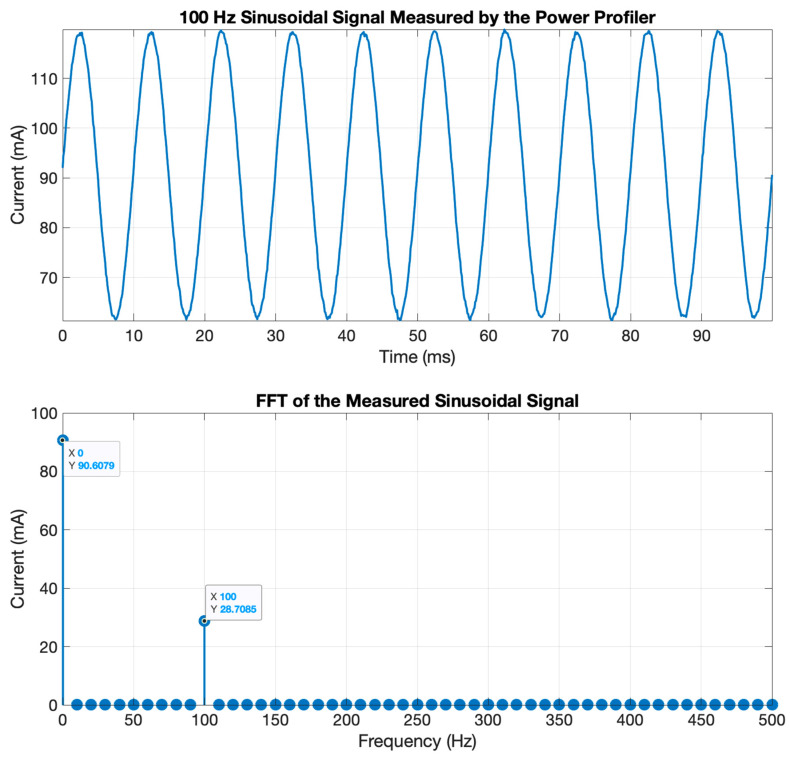
100 Hz sinusoidal signal used to determine the Power Profiler’s accuracy and its FFT.

BLE does not need a connection establishment per se, but when the device wakes up from a sleep mode, the modem needs to be reinitialised. This consumption (see Figure A10) must be taken into consideration every time the device sends a message: 51.98 mAs.

Thus, the full energy consumption for a 12-byte BLE transmission—considering both the modem initialisation and the message transmission—gets to be 52.99 mAs. For larger payloads, check Table 8.

## 4. Energy Harvesting Generation

The global increase in IoT nodes, and especially indoor ones, has led to the search for new ways of providing power supply to them. IoT devices are usually wireless, which means they need a battery (or other storage element) to operate. This battery needs to be replaced occasionally, which increase costs—in fact, the cost of replacing the battery can be greater than the IoT device itself. For exemplification, the device explained in Section 2 hereof has an average consumption of about 1 mW [5]. This device was wisely designed to be very power efficient, so it can run autonomously by means of MIEH.

Energy harvesting technology provides a green, carbon-free, sustainable, and virtually infinite power supply to wireless devices, obtaining the available energy from the environment to reduce—or even eliminate—the need for storage elements and wired power supply. Some of the most relevant EH methods for IoT devices are ambient radiation (RF), photovoltaic, piezoelectric, magnetic induction, vibration, pyroelectric and thermoelectric [5].

This paper is focused on the development and possibilities of what we consider the three main sources for indoor smart meters: photovoltaic (transforming light radiation into electrical current), radiofrequency (using the already-present electromagnetic waves) and magnetic induction (exploiting the changing magnetic fields that occurred in AC wires).

The nature of these three technologies entails a complex comparative study. In the literature, one can find different results for a bunch of designs, configurations and sets. Power generations depend widely on the real characteristics of the ambient energy availability, device’s design, system’s requisites, and implementation. However, it is crucial to compare these techniques with one another. Hence, for this work, we are using data from similar use cases found in the literature—comparing them by magnitude order instead of concrete values as they tend to be specific for each case. Be they:

- Photovoltaic: tens of milliwatts (10–20 mW)
- Radiofrequency: tenths of milliwatts (0.1–0.2 mW)
- Magnetic induction: milliwatts (1–2 mW)

### 4.1. Photovoltaic

Photovoltaic cells are used to power a multitude of sensors and other IoT systems. In [19], Xicai Yue et al. developed a PV EH model for a CO_2_ sensor, which produces an output of 4.2 V and a pulse current of 100 mA for 600 ms. They also determined that the best average storage efficiency is obtained at 200 lux. This is remarkable because, if a storage element is added to the PV harvester, the device could achieve its self-sustainability. Abhiman Hande et al. depicted in [20] the use of several ultracapacitors as energy storage devices. This leads to a compact and robust system with fewer solar panels in a series-parallel combination. 

The values of the indoor photovoltaic technique depend mainly on two criteria: light and material. Regarding the nature of light it usually would be artificial for inside use cases; in terms of material, the efficiency of new generation cells compared to commercial silicon is more than twice [21]. Nonetheless, it is important to note that real-world working conditions may drop theoretical efficiencies of PV panels to half [22].

Indoor lighting conditions are more complex than the exterior ones, since spaces are smaller, and the light levels are lower and intermittent. Indoor PV cells have a small size of around 30 cm^2^ [23]. A polymer-based silicon cell has a conversion power efficiency of 9% for F12 fluorescent lamps (100 lux). The light resource comes from natural outdoor light and artificial light from luminaires—fluorescent, LED. Solar cells require an irradiance of around 7 mW/m^2^ to work properly.

On the one hand, the non-constant presence of people reduces the daylight hours of the surroundings. On the other hand, average consumption is much lower than active consumption, saving energy and enabling this technology. The most common outdoor solar cells are made of silicon, a cheap material highly studied that produces 0.1 mW/lm. However, for indoor conditions where size requirements are tougher, researchers are studying new materials with larger bandgap energy, higher efficiency and smaller dark currents [24]. 

In [25], Lin Xie et al. use an organic photovoltaic cell for high intensity indoor environments, such as hospitals, with a theoretical efficiency of 60%. In the same way, the interest in gallium cells for indoor EH is also growing thanks to the lack of transparency losses, leading to a 40% energy conversion [22]. A small GaAs cell that is one-third of an office paper can power devices of up to 10 mW [26].

PV could be mixed with other technologies. For example, in [27] Yen Kheng Tan et al. create a hybrid of solar photovoltaic and thermal energy. The goal of this system is to extend the lifetime of the wireless sensor nodes. For a solar irradiance of 1010 lux and a thermal gradient of 10 K, an average of 621 W is obtained—three times more than just thermal. 

Indoor and outdoor uses can also be combined, for example for smart building applications. This is described in [28], where a PV EH generates an average of 0.5 W, getting to be a maximum of 2.3 W with 130 lux. 

### 4.2. Radiofrequency

Radio signals, mobile phones or Wi-Fi are some of the examples that originate these electromagnetic waves which are available almost everywhere. In addition, the fluctuations it presents are usually caused by human factor, not by weather conditions.

It is essential to consider the frequency, the modulation, and the power transmission to properly design the circuit of indoor devices. The key element is known as rectenna. Its main components are a rectifier and an antenna. In order to optimise energy conversion, a DC-DC boost converter can be added [29]. Many rectennas have been created, achieving different efficiencies depending on the design characteristics. 

The study of radiofrequency EH is related to the dBm value of the incident signal and the conversion efficiency. For –20 dBm, equivalent to 10 µW, the average efficiency may be around 10%, while if it is goes up to 0 dBm (1 mW), the efficiency could increase up to 50% [30]. 

In [31], a paper substrate rectenna designed for an input power of −20 dBm over LTE bands presented a range of 5–16% efficiency; while the six-band dual rectenna presented in [32] showed a range of efficiency between 37.7% and 41.4% with −15 dBm input power. In [33], A. Eid et al. presented an ultra-compact and flexible rectenna that can operate at the 2.4 GHz ISM band reaching up to 40% efficiency. 

For a 2.4 GHz Wi-Fi Multi-SSID router and power of 0 dBm, the energy harvester presented in [30] has an efficiency of 50.18%, due to the power management unit which minimise losses. Using the same circuit design, it is possible to operate on several ISM band or cellular RF as well [34]. 

Huaguang He et al. depicted in [35] a 2 × 4 rectenna array for the range of 895–925 MHz and 1.6–2.65 GHz. They obtained an output pulse voltage of 3.3 V and 33 mA for 100 ms, with an efficiency of 33% for 1800 MHz, 41.5% for 900 MHz and 55% for 2.4 GHz. However, it is important to note that the use of rectenna arrays at indoor environments might be inappropriate if the multipath effect is not eliminated since RF energy harvesting is sensitive to the angle of incidence, so adding more rectennas to the harvester is not a straightforward solution.

The current challenge is to improve the conversion capacity and energy harvesting of the rectennas. There are several possibilities, such as broadband antennas or antenna arrays. Nevertheless, the first requires a matching network for each frequency band and the second ones add complexity. 

### 4.3. Magnetic Induction

The third most relevant energy harvesting technique for indoor IoT smart meters or similar applications is magnetic induction. The basic requirement is to have a coil, either near or surrounding the wires carrying alternating current so that the change of inducted magnetic fields excite the coil, thus generating an electromotive force. This means that the electrical panel or distribution box must be opened, but no electrical disruption is needed as electrical circuits are not modified. This EH technique was studied and characterised in detail in our previous work “Smart Metering for Challenging Scenarios: A Low-Cost, Self-Powered and Non-Intrusive IoT Device”. In this paper [5], a metering IoT device was power supplied by the electromotive force generated inside the coil, providing around 1 mW of power for an average household mains line. 

When sensors or devices require current measurement, as in this case, MIEH is the optimal choice since it uses the same physical principle as current probes. In this way, the whole system is more compact, simpler, and cost-efficient. This appreciation of the use of magnetic induction as EH has hardly been developed and even less applying wireless IoT communications. The effective mains current energy harvesting range of this device is similar to that at home, corresponding to a current between 2.2 and 6.4 A. Using IEEE 802.15.4 as wireless technology, Danilo Porcarelli et al. achieved in [36] a device is self-powered with a load of 300 W for at least 60 s of measurement. 

## 5. Discussion

In this section, we assess the main challenge for autonomous indoor smart meters: power balance. We address the trade-off between working conditions, energy harvesting generation and wireless technology energy consumption.

The five wireless technologies in the scope are compared with different working conditions for the smart meter—metering period and buffer size—, at the same time they are evaluated to check if they comply with the available power generated by the EH techniques.

### 5.1. Wireless Technology Selection

After having characterised the five wireless technologies, we can now analyse the energy consumption each of them would have, and therefore discuss which one would be better depending on the power available.

This work was planned so that every wireless technology runs in the same development board. This allows us to not be dependent on the consumption of the metering process per se, being the same for all technologies. This was characterised in Section 2 hereof, and it is not worth considering for the wireless technologies comparison.

**Figure 6:**
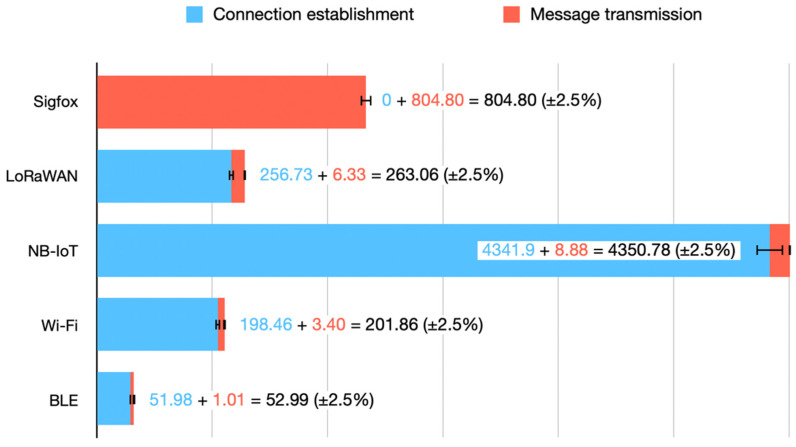
Quantitative representation of the wireless technologies’ consumption. A confidence interval of 5% is depicted as the set of measurements for every technology were within this range.

Figure 6 depicts a quantitative representation of the consumption needed for each technology—it is plotted out on a non-linear scale to enhance visualisation, since for the technologies requiring establishment, the vast majority of their consumption comes from that very process.

These consumptions are those from Table 3, where they were characterised for 12-byte messages as it is the Sigfox limitation. However, for some technologies, increasing the message payload does not proportionally increase the message transmission consumption—as one could see in Section 3, Table 4, Table 5, Table 6, Table 7 and Table 8.

This way, we could reduce the number of messages—thus lowering the number of connection establishments—, and therefore decrease the whole energy consumption. For instance, with LoRaWAN in our configuration (SF7, 125 kHz), we can send messages of up to 222 bytes. For the case of BLE, our board lets us send messages of up to 25 bytes. For the case of both Wi-Fi and NB-IoT, this limit is much higher and above 1024 bytes—768 bytes is the limit considered for comparison.

**Figure 7:**
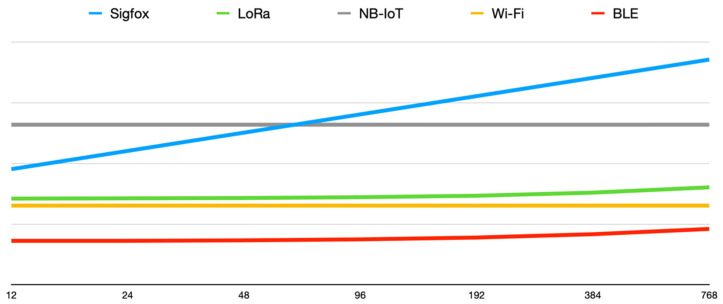
Plot of the wireless technologies’ consumption in logarithmic scale for different message sizes.

Table 9 shows the full consumption for each wireless technology and every message size. Moreover, in Figure 7 (logarithmic scale) we can see the evolution on the consumption required by each technology as the payload increases from 12 to 768 bytes.

Apart from the features of the communications—payload, communication establishment, number of transmissions—the characteristics of every use case will also determine the best suitable wireless technology for the application: what range should the device support, is there a strict real-time requirement for the data, can a public infrastructure be used, etc.

### 5.2. Energy Harvesting Technique Selection

For feasibility characterisation, not only the raw power available from the energy harvesting sources is important, but also their availability. For some EH sources, their availability may vary importantly whilst for others may be steadier, more predictable. For instance, indoor PV might be very different along time for the same space, as it will depend on the external weather, several people switching lights, and even the state of blinds. On the other hand, RF will be steadier along time as ambient radiation tends to be constant in the long term—it really only changes provided that new wireless technologies are deployed. MIEH may be also fairly predictable and constant, as the energy consumption of a building tends to be the same by periods—working days/weekends, working hours/nights, and holidays.

In order to avoid the lack of power supply, it could be possible to combine two types of EH techniques, such as PV and MIEH. However, this combination makes the device more complicated as two harvesting circuits would be required. Furthermore, the solar panel should be located outside the electrical panel to receive light, which adds complexity to the whole system. The same manner, if RF were used, the rectenna would also need to be outside the electrical panel to receive stronger signals.

The use of one EH source over another will depend on the specific use case: where will the device be located, will there be enough mains consumption to exploit MIEH, will there be ambient light to install a PV harvester, will there be much data to be transmitted, etc. Nonetheless, for smart meters the best approach gets to be MIEH: no elements need to be placed outside the electrical panel, and the same physical device acts as current probe—crucial for metering—and harvester, which simplifies the device, hence providing an easy, fast way of deploying ubiquitous smart meters.

Although adding more harvesters or using better ones could solve the power balance problem, this would also make the device more expensive—and complex if several harvesters are combined. Since this work is focused on smart meters, MIEH is the most appropriate solution in terms of scalability, simplicity and ease of installation; even if some trade-offs are faced using this technique, such as reducing metering period.

### 5.3. Constraints for Energy Consumption of Smart Meters

We can reckon the average power demand for every case with Equation (2). The result (Ψ) would be in mAs, which is directly converted to mW multiplying by the voltage supply of the characterisation (5 V).

This calculus let us write Table 10, where all possible combinations are shown, properly depicting the average consumption of the device. This is the consumption that must be counter-balanced by the EH source in order to make the device autonomous.

Considering EH generations as in Section 4 hereof, each table cell text is marked indicating the EH techniques that would comply to make the device autonomous—we consider a qualitative magnitude order, as claimed in Section 4:

- Circled: RF complies, so do MIEH and PV (never happens)
- Italics: MIEH complies, so does PV
- Underline: only PV complies
- Grey text: no EH technique would comply

(2) $$\Psi =\frac{\Sigma \times \Gamma +\Phi +\Sigma \times Τ\times \Theta }{\Sigma \times Τ}$$

where:

- Σ is the buffer size (bytes)
- Γ is the consumption of a standard metering process (mAs)
- Φ is the consumption of a transmission for the designated technology (mAs)
- Τ is the metering period (s)
- Θ is the stand-by consumption (mA)

The optimal combination of energy harvesting technique and IoT wireless technology will depend on functional requisites (metering period), communication requirements (buffer size) and location constraints (EH source availability).

Enlarging the stand-by periods of event-driven IoT nodes enhances power requirements, making them lower. However, this means losing time-precision in measurements because fewer samples are taken. This phenomenon could even lead to missing information about short events, such as the consumption peak that happened when heating something in a microwave oven, which may last for 3–5 min, but might be missed if the metering period is set to a higher value.

Nevertheless, enlarging the measurements buffer does not always enhance energy consumption. In the case of Sigfox, there is no difference at all since Sigfox’s consumption increases linearly as the buffer size increases, due to both its 12-byte payload limitation and the fact that it does not need any establishment consumption (see Table 4). The technology with the largest reduction in consumption by buffer enlargement is NB-IoT, mainly because it allows big payloads but requires a big establishment consumption; however, the transmission consumption per se is nearly the same with no regard to the message size (see Table 6). The others (LoRaWAN, Wi-Fi, BLE) present a restraint enhancement that, in most cases, will not be worth it as the data will update less frequently.

Moreover, as fewer measurements are taken—stand-by period extended, buffer size is increased, fewer wireless transmissions—the predominant consumption tends to be that coming from the smart meter per se, thus making wireless technologies consumption differences less noticeable in the overall average consumption of the IoT device. Notice that the consumption coming from the stand-by period and the metering process is the same regardless of the wireless IoT technology.

It is important to note that, with the development board and wireless technologies used for this work, radiofrequency energy harvesting cannot be used since the amount of energy that can be harvested is lower than the necessary to meet the autonomous working conditions.

## 6. Conclusions

Feasibility of autonomous indoor IoT smart meters is mainly restricted by the limitations on the ambient energy available to harvest. Although IoT devices have achieved very low power consumptions in the recent years, wireless technologies still require a considerably large amount of power to perform. With the aim of facilitating the design and development of autonomous wireless smart meters, we have performed a comparative analysis of the energy consumption of the trendiest IoT wireless communication technologies (Sigfox, LoRaWAN, NB-IoT, Wi-Fi, BLE), and the most appropriate EH techniques for smart meter applications (PV, RF, MIEH). This analysis will help smart meter developers, metering solutions providers and smart metering adopters in the selection of the most suitable combination of wireless communication technology and energy harvesting technique—based on their use case characteristics and location constraints.

Regarding IoT wireless technologies, it has been shown that the most energy efficient happens to be BLE, followed by LoRaWAN and Wi-Fi, finally tailing NB-IoT and Sigfox. However, their specific consumption will be determined by the metering period and buffer size. Customarily, BLE will be the best choice if communication range is not a limiting factor; otherwise, it will be LoRaWAN. However, if a commercial managed receiving network is wished, NB-IoT or Sigfox will be the suitable ones depending on data constrains.

Concerning EH techniques, the most powerful is PV, followed by MIEH, and finally RF. Yet, considering the smart meter field, MIEH gets to be the most appropriate since it takes advantage of the metering component (current sensor) as energy harvester, reducing device complexity and costs. Besides, smart meters are usually located inside electrical panels or distribution boxes—where no light is present.

Results prove that selection of wireless technology and energy harvesting technique will depend on the desirable application requisites—metering period, communication period—and the location constraints—EH source availability.

## Figures and Tables

**Table 1:** Summary for some critical characteristics of the IoT Wireless technologies.

Technology	Data Rate	Payload ^1^	Handshake
Sigfox	100 bps	12 B	No
LoRaWAN	5470 bps	222 B	Yes
NB-IoT	250 kbps	768+ B	Yes
Wi-Fi	16 Mbps	768+ B	Yes
BLE	1 Mbps	25 B	No

^1^ User-available payload per message/packet.

**Table 2:** Number of transmissions needed for each wireless technology for different payloads.

Technology	24-Byte	48-Byte	96-Byte	192-Byte	384-Byte	768-Byte
Sigfox	2	4	8	16	32	64
LoRaWAN	1	1	1	1	2	4
NB-IoT	1	1	1	1	1	1
Wi-Fi	1	1	1	1	1	1
BLE	1	2	4	8	16	31

**Table 3:** Summary of energy consumptions for IoT wireless technologies (12-byte messages).

Technology	MTC (mA)	ETC (mA)	Establishment Consumption (mAs)	Transmission Consumption (mAs)	Total Consumption (mAs)
Sigfox ^1^	<59.4	53.1	0	804.80	804.80
LoRaWAN	<59.4	62.5	256.73	6.33	263.06
NB-IoT	<277.2	141.2	4341.9	8.88	4350.8
Wi-Fi	118.8	109.9	198.46	3.40	201.86
BLE	85.8	95.9	51.98	1.01	52.99

^1^ Sigfox’s ETC is not calculated with the average consumption of the transmission process (Appendix A), but with the average current demand of one of the three transmissions occurred in Sigfox (90.1 mA), so as to not include the period between transmissions in the calculus.

**Table 4:** Summary of energy consumptions (mAs) for Sigfox.

Sigfox Payload (Bytes)	12	24	48	96	192	384	768
Establishment consumption	0	0	0	0	0	0	0
Number of transmissions	1	2	4	8	16	32	64
Transmission consumption	804.80	804.80	804.80	804.80	804.80	804.80	804.80
Total consumption	804.80	1609.6	3219.2	6438.4	12,876.8	25,753.6	51,507.2

**Table 5:** Summary of energy consumptions (mAs) for LoRaWAN.

LoRaWAN Payload (Bytes)	12	24	48	96	192	384	768
Establishment consumption	256.73	256.73	256.73	256.73	256.73	256.73	256.73
Number of transmissions	1	1	1	1	1	2	4
Transmission consumption	6.33	9.34	12.75	20.79	36.81	36.81	36.81
Total consumption	263.06	266.07	269.48	277.52	293.54	330.35	403.97

**Table 6:** Summary of energy consumptions (mAs) for NB-IoT.

NB-IoT Payload (Bytes)	12	24	48	96	192	384	768
Establishment consumption	4341.9	4341.9	4341.9	4341.9	4341.9	4341.9	4341.9
Number of transmissions	1	1	1	1	1	1	1
Transmission consumption	8.88	8.88	8.88	9.13	9.37	9.87	11.15
Total consumption	4350.8	4350.8	4350.8	4351.0	4351.3	4351.8	4353.1

**Table 7:** Summary of energy consumptions (mAs) for Wi-Fi.

Wi-Fi Payload (Bytes)	12	24	48	96	192	384	768
Establishment consumption	198.46	198.46	198.46	198.46	198.46	198.46	198.46
Number of transmissions	1	1	1	1	1	1	1
Transmission consumption	3.40	3.40	3.40	3.40	3.40	3.40	3.40
Total consumption	201.86	201.86	201.86	201.86	201.86	201.86	201.86

**Table 8:** Summary of energy consumptions (mAs) for BLE.

BLE Payload (Bytes)	12	24	48	96	192	384	768
Establishment consumption	51.98	51.98	51.98	51.98	51.98	51.98	51.98
Number of transmissions	1	1	2	4	8	16	31
Transmission consumption	1.01	1.01	1.01	1.01	1.01	1.01	1.01
Total consumption	52.99	52.99	54.00	56.02	60.06	68.14	83.29

**Table 9:** Summary of energy consumptions (mAs) for all message sizes for all technologies.

Technology	12-Byte	24-Byte	48-Byte	96-Byte	192-Byte	384-Byte	768-Byte
Sigfox	804.80	1609.6	3219.2	6438.4	12,876.8	25,753.6	51,507.2
LoRaWAN	263.06	266.07	269.48	277.52	293.54	330.35	403.97
NB-IoT	4350.8	4350.8	4350.8	4351.0	4351.3	4351.8	4353.1
Wi-Fi	201.86	201.86	201.86	201.86	201.86	201.86	201.86
BLE	52.99	52.99	54.00	56.02	60.06	68.14	83.29

**Table 10:** Smart meter average consumption (mW) depending on buffer size—i.e., message payload—, metering period and wireless technology.

Buffer Size (Bytes)	Metering Period (min.)	Sigfox	LoRaWAN	NB-IoT	Wi-Fi	BLE
**12**	**1**	15.07	11.31	39.69	10.89	9.85
	**5**	3.08	2.33	8.00	2.24	2.03
	**10**	*1.58*	*1.20*	4.04	*1.16*	*1.06*
	**15**	*1.08*	*0.83*	2.72	*0.80*	*0.73*
**24**	**1**	15.07	10.40	24.59	10.18	9.66
	**5**	3.08	2.15	4.98	2.10	2.00
	**10**	*1.58*	*1.11*	2.53	*1.09*	*1.04*
	**15**	*1.08*	*0.77*	*1.72*	*0.75*	*0.72*
**48**	**1**	15.07	9.95	17.03	9.83	9.57
	**5**	3.08	2.05	3.47	2.03	*1.98*
	**10**	*1.58*	*1.07*	*1.78*	*1.06*	*1.03*
	**15**	*1.08*	*0.74*	*1.21*	*0.73*	*0.71*
**96**	**1**	15.07	9.72	13.26	9.65	9.53
	**5**	3.08	2.01	2.72	*1.99*	*1.97*
	**10**	*1.58*	*1.05*	*1.40*	*1.04*	*1.03*
	**15**	*1.08*	*0.72*	*0.96*	*0.72*	*0.71*
**192**	**1**	15.07	9.60	11.37	9.57	9.50
	**5**	3.08	*1.99*	2.34	*1.98*	*1.97*
	**10**	*1.58*	*1.03*	*1.21*	*1.03*	*1.02*
	**15**	*1.08*	*0.71*	*0.83*	*0.71*	*0.71*
**384**	**1**	15.07	9.55	11.42	9.50	9.49
	**5**	3.08	*1.98*	2.15	*1.97*	*1.96*
	**10**	*1.58*	*1.03*	*1.12*	*1.03*	*1.02*
	**15**	*1.08*	*0.71*	*0.77*	*0.71*	*0.70*
**768**	**1**	15.07	9.52	9.95	9.50	9.49
	**5**	3.08	*1.97*	2.06	*1.97*	*1.96*
	**10**	*1.58*	*1.03*	*1.07*	*1.02*	*1.02*
	**15**	*1.08*	*0.71*	*0.74*	*0.71*	*0.71*

## Data Availability

Not applicable.

## Appendix A

This appendix contains current consumption graphs for every wireless technology characterised and the smart metering process.

## Appendix B

This appendix contains information about the process used to determine the Power Profiler’s metering accuracy.
